# Supplementary material for: A Critical View of the Application of the APEX Software (Aqueous Photochemistry of Environmentally-Occurring Xenobiotics) to Predict Photoreaction Kinetics in Surface Freshwaters
Source: Molecules. 2019 Dec 18;25(1):9. doi: 10.3390/molecules25010009 (PMC7017383; doi:10.3390/molecules25010009)
Supplement: Supplementary file 1 [file molecules-25-00009-s001.zip › 3.2.4_gcc-4.4.0.zip!/3.2.4_gcc-4.4.0/3.2.4_gcc-4.4.0/share/octave/packages/audio-1.1.4/doc/endpoint.doc]

Interactive speech recognition systems are only useful if they can

can run with live input. The problem with live input, as opposed to

pre-recorded data, is that the exact start and end of the utterance

is unknown. One technique to deal with this problem, is to record

a fixed size utterance (e.g., 5 seconds) and assume that the user will

speak the entire utterance within the time period. A recognizer which

has silence models for the start and end of the utterance can thus

parse such an utterance. However, such a scheme is obviously prone

to errors and is computationally wasteful because the entire input

buffer must be searched.

The obvious solution is an endpointer which identifies the start and

end of utterance. The problem is that endpointing an utterance, like

speech recognition itself, is non-trivial.

This is an endpointing algorithm designed for real-time input of

a speech signal. "Real-time" means that the signal is processed

in parallel with its recording. This allows a speech recognition

system to run in parallel with the input of the utterance.

This algorithm calculates and uses "cheap" parameters, RMS energy and

zero crossing counts. Thus, this algorithm can run in real-time on

any micro processor without the need for a DSP.

Because the signal is end-pointed in real-time, errors can and do

occur in identifying the start and end of the actual utterance.

Thus, the labels, or tags, that this endpointer gives for each

frame of data are some what "fuzzy". That is, the endpointer will

tentitively label a frame but may indicate at a later frame that

the identification of a previous frame was in error. This requires

special handling by the speech recognition system in that it must

be capable of re-starting recognition after false starts and continuing

searching after possible end of utterance frames.

The endpointer works by passing to it one frame of data at a time. The

endpointer will check the frame to determine if it is part of the

utterance and return a label, or tag, for the frame. The possible

labels are the following:

EP_NONE

EP_NOSTARTSILENCE

EP_SILENCE

EP_SIGNAL

EP_RESET

EP_MAYBEEND

EP_NOTEND

EP_ENDOFUTT

EP_NONE - This is a NULL label which the endpointer does not return,

This is convenient to have for labeling frames for which the endpointer

is turned off.

EP_NOSTARTSILENCE - The first frame is so loud or noisy that it does not

"look" like background silence. This depends on absolute thresholds and

can generate a false positive for really noisy signals or a false negative

for really quiet signals. See theory of operation below.

EP_SILENCE - This label is returned for silence frames before the start

of the utterance.

EP_SIGNAL - This is returned for each frame that appears to be contained

in the utterance signal. The first E_SIGNAL frame marks the start of the

utterance.

EP_RESET - This indicates a false start condition. The previous EP_SIGNAL

frames were, in fact, not part of the utterance. The recognition system

should reset itself and start over.

EP_MAYBEEND - This label indicates the possible end of utterance. The

frame which has this label is actually one frame after the possible last

frame of the utterance. As this is a tentative label, the recognition

system should either do end of utterance processing or save its state at

this point for end of utterance processing. In either case, the recognition

system must continuing searching, including this frame, until the end of

utterance has been confirmed.

EP_NOTEND - The previous EP_MAYBEEND label was wrong. The utterance is

continuing. The recognition can now forget its possible end of utterance

state.

EP_ENDOFUTT - The label confirms the actual end of utterance. The real

end of utterance was the last EP_SIGNAL frame before the last EP_MAYBEEND

labeled frame.

Theory of operation:

For each frame of data, the endpointer calculates the RMS energy and the

zero-cross count. The first few frames are assumed to be background

silence and are used to initialize various thresholds. If there is no

starting silence (the user speaks too soon), then the endpointer will

mislabel the first syllable (which may be one or more words) until a

silence is reached. Similarly, if there is no ending silence, then the

endpointer will not mark the end of utterance.

A running average of the background silence is kept which consists of

averaging the last few silence frames. This background silence is used

to set energy thresholds and the Schmidt trigger for the zero-cross

counter.

The endpointer contains over a dozen thresholds and settings which are used

to determine frication, voicing, and silence. These thresholds have been

determined emperically.

The sampling rate, window size in samples, and the step size in samples

are passed to the class constructor. These three arguments are used to

calculate the internal thresholds (actual zero-cross count values for

frequencies and number of frames for durations). Any or all of the internal

CAVEATS:

The endpointer will fail if there is no starting silence or endsilence.

If there is no starting silence, then the first syllable up to the first

stop consonant will be lost. If there is no ending silence, then the last

syllable will the lost or no end of utterance will be determined.

thresholds can be changed by specifying them in the class constructor.

The endpointer makes no distinction between noise and speech. Impulse

noises will fool it. The endpointer tends to be conservative in that it

will err by including noises with the signal rather than cutting out part

of the actual speech signal. So, a good recognition system must model

noise.

Large amplitude background white noise may cause the endpointer to miss

fricatives, weak or strong. If the background noise is known a priori, then

the endpointer thresholds can be adjusted to cope with the noise.
